# Gross motor coordination in relation to weight status: a longitudinal study in children and pre-adolescents

**DOI:** 10.3389/fpubh.2023.1242712

**Published:** 2023-12-14

**Authors:** Valentina Biino, Barbara Pellegrini, Chiara Zoppirolli, Massimo Lanza, Federica Gilli, Matteo Giuriato, Federico Schena

**Affiliations:** ^1^Department of Neuroscience, Biomedicine and Movement Science, University of Verona, Verona, Italy; ^2^Department of Human Science, University of Verona, Verona, Italy; ^3^CeRiSM, University of Verona, Rovereto, Italy; ^4^Department of Engineering for Innovation Medicine, University of Verona, Verona, Italy

**Keywords:** KTK, longitudinal, BMI, children, pre-adolescence

## Abstract

**Introduction:**

Gross Motor Coordination (GMC) is crucial for the adequate development of motor competence. Our purpose in this semi-longitudinal study was to evaluate the influence of BMI on GMC in children and pre-adolescents of both sexes, across school years (classes).

**Methods:**

We evaluated 117 subjects (aged 8–13 years) belonging to three different cohorts for 4 consecutive years, providing data over 6 years (classes). GMC was assessed through the Körperkoordinationstest für Kinder (KTK) test. Class and weight status effects were then evaluated by dividing the subjects into a normal weight group and an overweight group based on their weight status.

**Results:**

A significant increase across classes was found for BMI (*p* < 0.001) and KTK raw score (*p* < 0.001) and a decrease was found for KTK normalized score (MQ) (*p* = 0.043). Significantly lower MQ values were found for girls. Absolute GMC increased across the years and there was no difference between boys and girls. Correlations between GMC scores and BMI were negative and significant in 5 of 6 classes. It was confirmed that overweight subjects had lower MQ and RAW values than normal-weight subjects, with no class-by-weight status interaction.

**Discussion:**

The level of competence and its development are strictly dependent on weight status during childhood and pre-adolescence. The present investigation suggests that the adequate development of GMC requires not only targeted physical education programs but also the promotion of healthy habits aimed at maintaining a normal weight status during childhood and pre-adolescence.

## Introduction

Motor coordination has been associated with the individual ability to perform a variety of motor actions that require gross and fine motor coordination [(1), p. 95], from high-level sport-specific actions (2) to everyday life situations (3). The repertoire of gross motor coordination (GMC) includes different fundamental locomotor skills. The word *fundamental* was recently explained by Newel (4) as meaning *basic* rather than *important.* Fundamental motor skills, thus, include walking, running, leaping, jumping, hopping, skipping, galloping, sliding, the capacity to maintain balance and body stability, task control, and fundamental manipulative skills such as throwing, catching, kicking, trapping, striking, volleying, bouncing, and ball rolling [(5–9); Gallahue and Goodway, 2012].

The foundational skills required for being a proficient mover are found in mastery of locomotor movements, basic manipulative skills, and stability. Fundamental motor skills learning begins from the age of 2 years and develops rapidly, achieving higher rates of development during childhood and pre-adolescence (10, 11). However, it is also clear that low levels of physical activity, a significant decrease in motor experiences (12), and poor learning environments (13, 14) can lead to impairments or declines in gross (10, 11, 15, 16) and fine (16, 17) motor coordination in school-aged children. It is far from being proven that skill levels naturally reach the standards that encourage or facilitate participation in recreational, sporting, or motor activities (18). The level of physical activity engagement significantly affects the rate of gross and fine motor skill development (5, 19–21).

The interaction between actual competencies and perceived competencies promotes physical activity engagement in individuals (22). Adequate motor competence during childhood was suggested to represent a necessary base for the further development of superior motor abilities required to participate in more complex physical and motor activities or sports (2, 23). In the past decade, an increasing interest in exploring the relationships between GMC and weight status, from early childhood to adolescence emerged (24). The data available in the scientific literature suggested that children and adolescents with overweight, obese, or under-weight status show a lower level of GMC with respect to normal-weight peers, regardless of age (25, 26). Excluding underweight children, negative relationships between weight and GMC levels were also found (27–29).

However, the majority of the literature concerning GMC development during childhood and adolescence is principally based on cross-sectional studies that proposed different protocols, evaluations, and approaches to GMC assessment, thus limiting the global understanding of the matter (17). For example, some cross-sectional studies have shown the development of balance skills only (30, 31), while others evaluated the development of strength-related gross motor skills across the years (16), with no possibility to assess eventual causal relationships among parameters. Only longitudinal studies can facilitate a precise evaluation of the parameters that develop from childhood to adolescence, such as body mass and height (32), or parameters related to motor competencies (5, 10, 33). For example, basal ganglia and cerebellum, which are two important structures of the brain involved in the motor system (34), experience significant structural and functional changes across childhood and adolescence, having a significant impact on individual GMC development. To the best of our knowledge, the longitudinal studies already present in the literature deal with the correlations between physical activity and BMI (35) or assess physical activity from childhood to adulthood (36).

Thus, it appears evident that longitudinal studies could help to provide new consistent information about GMC evolution in relation to age, sex, and BMI (33). To this aim, we recruited school-aged boys and girls from 8–9 to 13–14 years of age, for 4 consecutive years; their GMC was evaluated through the KTK test, which is suggested as a reliable tool for longitudinal GMC measurements (37). This analysis has three specific aims: (i) to investigate GMC and BMI evolution in boys and girls, (ii) to assess the influence of BMI on GMC levels at each of the six developmental stages observed, and (iii) to evaluate eventual differences in GMC trends in normal weight and overweight children across the years. We hypothesized an increasing trend for the absolute values of GMC (Kiphrad and Schilling, 2007) and BMI (38) over time, in accordance with the literature (10, 11, 16). Moreover, we expected that the condition of being overweight would influence GMC negatively within each class in accordance with non-longitudinal studies, with overweight subjects performing worse than normal-weight subjects. However, here, we investigate for the first time whether overweight children from the first to the last class had different trends in GMC and BMI with respect to normal-weight children, with the hypothesis that the condition of being overweight would limit GMC development.

## Materials and methods

### Participants and study design

The present study analyzes data from a sample of 117, 57 boy (M) and 60 girl (F) subjects from the Northern part of Italy. The children and pre-adolescents belonged to two different primary schools (PS) (from class 1 to 5 in Italy) and secondary schools (SS) (from class 1 to 3 in Italy) in the Veneto region. They were taken from three different cohorts and tested for the first time when they were in the 3rd, 4th, and 5th classes of PS (3PS, 4PS, 5PS) for cohorts 1, 2, and 3, respectively. They all were evaluated in the month of February for 4 consecutive years, between 2017 and 2020. The number of subjects in each cohort and class and a descriptive statistic of each class (indicating chronological age, height, weight, and BMI (mean ± 1 SD) are presented in Table 1.

**Table 1:** Description of cohort composition and children’s characteristics for each class analyzed [chronological age, height, weight, and BMI (mean ± 1 SD)].

		3 PS	4 PS	5 PS	1 SS	2 SS	3 SS
Subjects (*N*)	*Cohort 1*	30	30	30	17		
	*Cohort 2*		44	44	44	44	
	*Cohort 3*			43	43	43	43
	*Total*	30	74	117	104	87	43
	*F*	11	37	57	51	47	20
	*M*	19	37	60	53	40	23
Age (yrs)	*Mean*	8.1	9.2	10.2	11.1	12.0	12.9
	*(SD)*	(0.3)	(0.4)	(0.4)	(0.4)	(0.3)	(0.3)
Height (m)	*Mean*	1.30	1.36	1.43	1.49	1.56	1.63
	*(SD)*	(0.07)	(0.07)	(0.08)	(0.09)	(0.09)	(0.09)
Weight (kg)	*Mean*	29.8	35.2	40.2	45.2	49.9	53.6
	*(SD)*	(5.6)	(8.2)	(10.0)	(11.2)	(12.0)	(13.0)
BMI (kg/m2)	*Mean*	17.7	18.9	19.4	20.1	20.5	20.1
	*(SD)*	(2.6)	(3.4)	(3.8)	(3.9)	(4.2)	(4.2)

F, girl; M, boy; SD, standard deviation; PS, primary school; SS, secondary school; BMI, Body Mass Index.

Moreover, the weight status of each subject was classified using the age- and sex-specific BMI cut-off points for children proposed by the International Obesity Task Force (38). According to this categorization, two types of subdivision were considered for the present sample of subjects. Firstly, subjects were divided into two groups, a normal weight (NW1) group and an overweight (OW1) group, considering their weight classification at the first evaluation. Secondly, only the subjects that maintained their weight status from the first to the last evaluation were considered and were divided into a normal weight (NW2) group and an overweight (OW2) group. This subdivision allowed an additional analysis of GMC development among children and pre-adolescents.

### Materials and procedure

Children’s anthropometric characteristics as well as GMC levels were always evaluated using the same experimenter (their teacher, who was also responsible for the research) in the school gymnasium, during physical education classes. During the evaluations, participants wore sportswear and sports shoes, to avoid the risk of slipping, which they would usually wear during scholastic physical activity.

The parents (or legal guardians) of each child gave their written consent for participation, after having received a detailed written explanation about study procedures and possible risks. Study protocol and data collection were conducted in accordance with current national and international laws and regulations governing the use of human subjects (Declaration of Helsinki II). The Ethics Committee approved the study (N. UNVRCLE-0298910).

The BMI of each subject was calculated as the ratio between weight (kg) and squared height (m^2^). Body height and weight were determined by means of a metric string with 0.1 cm resolution and a digital scale with 0.1 g resolution, respectively.

The GMC level was evaluated using the standardized and validated battery of tests, the KTK (39). Although some authors have suggested the use of the short version of KTK (including 3 items), we decided to use the originally proposed 4-element version, considering that the elimination of the only item involving the use of the upper limbs could lead to an incomplete assessment of coordination ability (40).

The 4-element KTK version included:

(1) *Walking backward (WB)* three times on balance beams with progressively lower width: 6.0, 4.5, and 3.0 cm. The number of steps taken when going backward was counted up to 8 steps for each beam or until one foot left the beam. This allowed a maximum score of 72 (3 × 3 × 8).
(2) *Moving sideways (MS)* on standardized boxes for 20 s, over two separate trials. Each trial consists of moving the boxes sideways and jumping on them. The child stands on the box placed on the right, takes the box on the left with both hands, and puts it on the floor again on their right-hand side; they then jump on it and pick up the box on their left, and so on. The changing of the direction of the boxes was done from left to right or vice versa, according to the child’s preference, and was maintained in the two attempts. The total number of relocations was summed to get the final score.
(3) *One-legged jumping (HH)* over obstacles consisting of 5 cm thick foam cushions, which are stackable to increase height (maximum 12 cushions). For each height jumped, 3, 2, or 1 point was awarded for a successful jump on the first, second, or third attempt, respectively. The maximum score obtainable with 12 panels (height 60 cm) was, therefore, 39 points for each leg and 78 total points.
(4) *Jumping sideways (JS)* across a standardized wooden slat with both legs for 15 s; the number of jumps over two trials was summed.

For each KTK item (WB, MS, HH, JS), a score was assigned following the test instructions (37, 39). The raw score (RAW) was calculated by adding together the scores of the four items. Each item score was also normalized (accounting for subject sex and age) using the specific tables of conversion proposed by the authors (37, 39), which were based on an algorithm extrapolated by their empirical observation. All the normalized scores were then added together to obtain a global normalized score (Motor Quotient, MQ), expressed as a numerical value (with the range between 85 and 115 considered “normal”). The KTK test had already been demonstrated to be a valid and reliable tool to assess GMC. The test–retest for the RAW on the total test battery had a reliability coefficient of 0.97, while specific coefficients for each subtest ranged from 0.80 to 0.96 (37, 39).

### Statistical analysis

Since subjects belonging to the different cohorts were not tested in the same time intervals (classes), linear mixed models for repeated measurements (LMMRM) were used to examine the effect of classes and BMI on GMC indexes. The advantage of utilizing linear mixed models for the analysis of longitudinal data extends to their robustness in dealing with missing data, particularly when the missingness is independent of both unobserved and observed data. The within-subject nature of the data was accounted for by including classes as a fixed within-subject factor and by modeling residual errors with a first-order autoregressive covariance structure, under the assumption that correlation within subjects is higher in adjacent classes and decreases over time (classes).

To test aim (i), which analyzed GMC and BMI evolution in boy and girl subjects, the statistics were calculated using a two-factor LMMRM model, using class and sex as fixed factors on RAW, MQ, and BMI. To test aim (iii), which evaluated GMC trends in normal-weight and overweight children across years, the statistics were calculated using a two-factor LMMRM model, using class and weight status as fixed factors on RAW Score and MQ. *Post hoc* analysis was run, including *Bonferroni*’s correction to control for Type I error. To test aim (ii), which assessed the influence of BMI on GMC level, *Spearman’s rank* correlations test was used to evaluate correlation coefficients and statistical significance. All the statistical analyses were conducted using SPSS 22.0 (SPSS, Inc.; Chicago, Illinois). The level for statistical significance was set at *p* = 0.05.

## Results

BMI increased across observations (Table 2). No significant sex effect (*p* > 0.05) was found, while a class x sex effect was verified (Table 2). The *post hoc* test showed a significant increase in BMI in adjacent classes between 5PS and 1SS and between 1SPS and 2SS (*p* < 0.001), in boy subjects only. Differences between boy and girl subjects were found in 4PS and 5PS. Weight and height showed a significant class effect, with a significant increase in every class with respect to the previous. No significant effect was found for either class or sex and no interaction between these two factors on weight and height was found.

**Table 2:** Rawscore and Motor Quotient of the KTK, BMI, height and weight in the different classes and sexes.

			3 PS	4 PS	5 PS		1 SS		2 SS	3 SS	*Class*	*Sex*	*Class × Sex*
			*N* = 30	*N* = 74	*N* = 117		*N* = 104		*N* = 87	*N* = 43			
Raw score	F	*Mean*	196.00	208.73	224.82		238.31		242.66	241.35			
		*(SD)*	(22.88)	(25.21)	(22.32)		(22.22)		(23.73)	(23.16)	*F* = 35.210	*F = 0.859*	*F = 0.870*
	M	*Mean*	193.53	214.49	228.98		238.15		246.53	254.70	*p* < 0.0001	*p = 0.320*	*p = 0.501*
		*(SD)*	(24.03)	(22.15)	(27.56)		(24.48)		(30.29)	(33.34)			
				§§	§§		§						
MQ	F	*Mean*	102.18	94.95	96.19		99.63		96.43	91.30			
		*(SD)*	(10.96)	(10.95)	(9.89)		(10.52)		(12.89)	(11.74)	*F = 2.318*	*F = 5.744*	*F = 1.851*
	M	*Mean*	102.74	102.76	100.65		101.36		100.35	99.78	*p = 0.043*	*p = 0.018*	*p = 0.102*
		*(SD)*	(11.61)	(9.90)	(12.31)		(11.28)		(14.33)	(16.44)			
				**	*								
BMI (kg/m^2^)	F	*Mean*	17.43	19.34	20.25		20.61		20.89	21.24			
		*(SD)*	(2.02)	(3.73)	(4.03)		(4.21)		(4.43)	(4.10)	*F = 7.953*	*F = 2.698*	*F = 2.434*
	M	*Mean*	17.81	18.40	18.67	§	19.66	§§	19.98	19.08	*p < 0.0001*	*p = 0.102*	*p=0.035*
		*(SD)*	(2.90)	(2.97)	(3.40)		(3.61)		(3.83)	(4.05)			
				*	**								
Height (m)	F	*Mean*	1.31	1.36	1.43		1.49		1.55	1.61			
		*(SD)*	(0.09)	(0.09)	(0.08)		(0.09)		(0.07)	(0.05)	*F = 174.1*	*F = 0.526*	*F = 2.224*
	M	*Mean*	1.29	1.36	1.43		1.49		1.56	1.65	*p < 0.0001*	*p = 0.469*	*p = 0.051*
		(SD)	(0.05)	(0.06)	(0.08)		(0.09)		(0.11)	(0.11)			
				§§	§§		§§		§§	§§			
Weight (Kg)	F	*Mean*	30.01	36.38	42.07		46.30		50.53	54.50			
		*(SD)*	(6.09)	(9.55)	(10.65)		(11.41)		(11.89)	(10.49)	*F = 80.69*	*F = 1.030*	*F = 1.454*
	M	*Mean*	29.71	34.06	38.43		44.21		49.23	52.79	*p < 0.0001*	*p = 0.311*	*p = 0.204*
		*(SD)*	(5.41)	(6.57)	(9.18)		(10.97)		(12.20)	(15.06)			
				§§	§§		§§		§§	§§			

This table illustrates Rawscore (RAW), Motor Quotient (MQ) and BMI across the classes in girls (F) and boys (M). On the right part of the table, class, sex and classXsex effects are specified.

SD, standard deviation; PS, primary school; SS, secondary school; *significantly different from F with *p* < 0.05; **significantly different from F with *p* < 0.01. §, significantly different from previous class with *p* < 0.05; §§, significantly different from previous class with *p* < 0.01. When a classXsex interaction was detected, differences between classes were assessed separately for boy (M) and girl (F) subjects, with the symbol § placed on the corresponding line. If no interaction was found, the symbol § was placed on the line below the respective M and F values.

GMC scores for different classes and sexes are reported in Table 2. A significant class effect was found for RAW and MQ. *Post hoc* analysis showed a higher score for RAW in 5PS, 1SS, and 2SS with respect to the previous class (Table 2). Moreover, the MQ score exhibited a significant sex effect, with boys outscoring the girls (Table 2).

**Figure 1:**
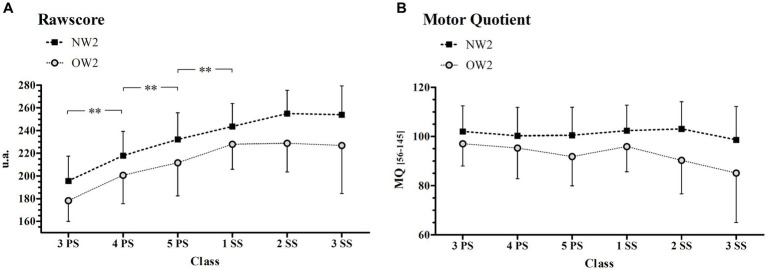
Spearman’s rank correlation between Ra0wscore/MQ and BMI. This figure illustrates the Spearman rank correlation between Rawscore and BMI and between Motor Quotient (MQ) and BMI across the classes. In each panel, *r* represents the Spearman’s correlation coefficient while the asterisks represent the level of statistical significance: *p < 0.01; **p < 0.001. PS, primary school; SS, secondary school.

Spearman’s rank correlations between BMI and RAW and between BMI and MQ values are presented in Figure 1 for all the evaluated classes considering boy and girl subjects together (since no sex effect was previously found concerning BMI values in a normal population). All the correlation coefficients were negative and varied between −0.19 and −0.40, indicating higher GMC levels associated with lower BMI values. However, the significance of the correlation was verified from 4PS to 2SS, both for RAW vs. BMI and for MQ vs. BMI, with the highest correlation being recorded at 2SS.

**Figure 2:**
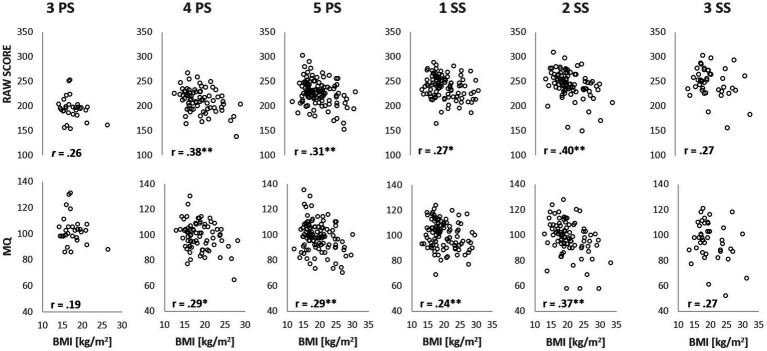
Gross motor competence in Normal weight (NW1) and Overweight (OW1) group. RAW **(A)** and MQ **(B)** trends in NW1 and OW1 across the classes. NW1, subjects showing a normal weight status at the first evaluation; OW1, subjects showing an overweight status at the first measurement; PS, primary school; SS, secondary school; *Significantly different from the previous class with *p* < 0.05; **Significantly different from the previous class with *p* < 0.01; #, Significantly different from OW1 with *p* < 0.05.

Looking at the subject subdivision accounting for the weight classification at the first evaluation (NW1, 76 children; OW1, 41 children), the effect of weight status and class was studied (Figure 2). Results of LMMRM analysis showed significantly higher RAW (*p* = 0.002, *F* = 10.48) and MQ (*p* = 0.004, *F* = 8.42) (Figures 2A,B, respectively) for the NW1 group than for the OW1 group. Moreover, the RAW showed a significant class effect (*p* < 0.001; *F* = 29.71) with increased GMC levels from 3PS to 1SS (*p* < 0.001). No significant interactions occurred (*p* > 0.05) for RAW, and no class or interaction effects were found for the MQ value (*p* > 0.05).

**Figure 3:**
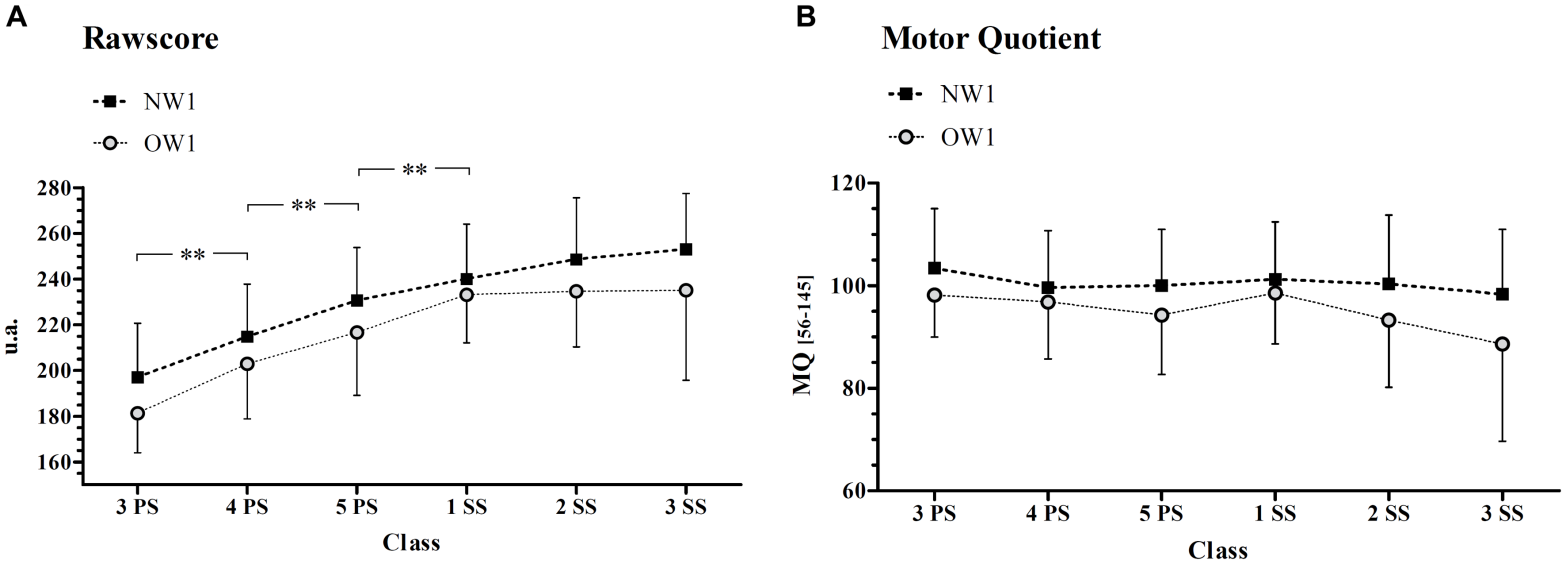
Gross Motor competence in Normal (NW2) and Overweight (OW2) group. Rawscore **(A)** and Motor Quotient **(B)** in NW2 and OW2 groups across the classes. NW2, subjects maintaining a normal weight status from the first to the last evaluation; OW2, subjects maintaining an overweight status from the first to the last evaluation; PS, primary school; SS, secondary school; **Significantly different from the previous class with *p* < 0.01.

Considering only the children who maintained normal weight or overweight status from the first to the last evaluation (83 over 117 children, NW2 = 61; OW2 = 22), the effect of weight status and class was examined (Figure 3). A significant effect of weight status was found on RAW (Figure 3A) and MQ (Figure 3B) (*p* < 0.001; *F* = 21.62 and *p* < 0.001; *F* = 16.13 respectively), with the OW2 group performing significantly worse than NW2. A significant class effect was found for the RAW score (*p* < 0.001; *F* = 22.80) but not for MQ (*p* = 0.185; *F* = 1.516). There was no interaction effect between classes and weight status for both RAW (*p* = 0.596; *F* = 0.737) and MQ (*p* = 0.312; *F* = 1.195). *Post hoc* results for class effect in the RAW score showed a significantly higher value in class 4PS, 5PS, and 1SS with respect to each previous class (Figure 3A).

## Discussion

The principal aim of the present study is to analyze GMC and BMI trends in young people between 9 and 14 years old, following a semi-longitudinal observational approach. The present research also aimed to evaluate the relationships between GMC level and BMI across the classes analyzed, as well as to track the evolution of GMC in normal-weight and overweight children and pre-adolescents across classes. The main results were as follows: (i) when considering the entire sample of subjects, BMI and RAW trend increased across classes without a sex effect, whereas the MQ score slightly decreased, with the boys outscoring the girls; (ii) we found an inverse relationship between GMC indexes and BMI in the majority of the classes evaluated here, children with a higher level of GMC showing lower BMI; and (iii) when considering two different subdivisions between normal-weight and overweight subjects (according to weight status at the first evaluation or longitudinally), we observed that overweight subjects always performed significantly worse than normal-weight subjects, at all ages.

This longitudinal analysis revealed that children’s BMI increased across classes, particularly in boy subjects of adjacent classes between 5PS and 2SS, ranging on average from the 50th to the 85th percentile of the age-specific standards set by the WHO (41). We also found an increasing trend in RAW score when considering the entire sample of subjects, highlighting increased GMC competence across classes, without significant sex differences. In this case, the *post hoc* revealed a significant increase in RAW scores in adjacent classes between 4PS and 2SS, while a stagnating situation between the last two classes was observed. It was proposed that GMC can develop significantly when adequate engagement in physical activity is maintained (13), determining whether a child will thrive in competence due to the learning environment (42). From an ecological perspective, skilled movement can be considered when conscious control of degrees of freedom can be achieved within the human system (43). Different environmental situations and performers’ perceptions of environments can alter how a performer successfully achieves a task (3). This ability depends on a vigilant sensory organ that dictates human movement choices. Although few GMC assessments provide dynamic perceptual contexts, the KTK evaluates using factor analysis, which points to a substantial homogeneity in the test tasks and the movement dimension “total body control,” and can be used to evaluate its evolution. It is also appropriate for young disabled people. An emotionally unstable child is instructed with varied wording so that they can gain some confidence and lose their fears. The test leader is consequently given a certain leeway when communicating with disabled children. However, it is important that information is transmitted completely to each child and that only the way in which the information is given varies with the aim of identifying the child’s optimal limit of output (39).

The normalized score slightly decreased across classes; however, it was maintained within a range of normality (i.e., MQ between 85 and 115 points) in each of the specific classes evaluated here. This was in agreement with a previous investigation (44) and suggests that children and preadolescents involved in the present study decrease in motor competence across classes compared to their German and Belgian peers used as a reference for the KTK normalization. Consistent with this, some authors (45) have suggested that an appropriate approach and motor competence since early childhood positively influences motor coordination during adolescence. RAW scores showed a significant difference between sexes, with boys slightly outperforming respect to girls. The meaning of this result is that the girls in our research sample were less skilled than boys, considering the score of the general population. Previous research has revealed sex differences in fundamental motor skills such as locomotive movements, balance, and manipulative movements. Boys were shown to be better performers in tests assessing manipulative movement skills (46), whereas girls were found to perform better in tests assessing balance (47).

The results of the present investigation should also depend on differences in physical activity engagement, even though we did not investigate this in the present study. Tonge et al. (48) showed that young girls are usually less engaged in physical activity than boys. This could be partially compensated by the observation that being a girl is associated with a lower relative risk of obesity (49). Jaakkola et al. (50) also proposed that the decrease in girls’ motor competence was linked to a decrease in their opportunities for physical activity.

A particularly important result of the present study was that the correlations between GMC and BMI were significant and negative in all the classes, except for the first and last classes evaluated here (3 PS and £ SS, respectively), indicating that higher GMC levels are associated with lower BMI values. D’Hondt et al. (51) found that a child’s BMI was a significant predictor of KTK (GMC) performance, indicating that a higher BMI is likely to be associated with poorer motor coordination. This relationship was observed in a sample of 100 children aged 6–10 years. Viceversa et al. (20, 21) found that a child’s level of GMC also predicts changes in subcutaneous adiposity (fat accumulation), with children with better KTK performance showing a smaller increase in subcutaneous adiposity over time. This suggests that a child’s weight status can influence motor competence and vice versa. The strength of the present study is that it supports and reinforces previous observations, having found that the correlations between GMC and BMI are also significant within single classes.

These considerations are also connected with the results of the third aim of the present study, which showed, for the first time, how GMC development differs between normal-weight and overweight subjects during childhood and pre-adolescence. When dividing normal-weight and overweight children on the basis of their weight status at the first evaluation, we found similar GMC trends across classes. However, we found that normal-weight children (NW1) performed better than overweight children (OW1) in GMC performance, both for RAW and MQ indexes: the absence of significant interaction effect between classes and weight status suggested that, on average, a child that is overweight during childhood cannot fill the gap in GMC levels throughout the years. Furthermore, it should also be considered that the most crucial ages to improve GMC is late childhood (52).

Very similar results for GMC trend and weight status differences were found when considering only the subjects that maintained longitudinally their normal weight (NW2: approximately 70% of the sub-sample) or overweight status (OW2: approximately 30% of the sub-sample). Indeed, OW2 classes showed a GMC level at the lower limit of normality (Normal MQ: 85–115), with an MQ score below 85 considered problematic since it represents less than the 15th percentile (37). It was previously demonstrated (11) that ectomorphic children show the best GMC development across time. On the other hand, our data showed that childhood obesity or being overweight is a reasonable predictor of adolescent obesity, which is in line with a review by Simmonds et al. (53).

In this regard, Malina et al. (54) found that a surplus of body mass negatively influences some motor tasks such as running and jumping. The current findings in this area of research explain that GMC is lower in overweight subjects than in normal-weight subjects since children’s skills should be influenced by the additional mass that needs to be supported or moved during motor tasks (55). Moreover, overweight and obese children also showed worse levels of fine motor performance (56), hindering these children from performing normal daily activities. To date, it has been demonstrated that GMC may be considered an important condition for engagement in organized physical activities; motor skills and coordination determine subsequent sports participation. Finally, it was proposed that overweight children have lower self-concept perceptions regarding their physical abilities compared to normal-weight children. This suggests that being overweight can impact children’s perception of their own physical capabilities, suggesting that children’s self-concept perceptions accurately reflect their actual physical abilities (57).

## Conclusion

The results of this longitudinal study, which explores data across six classes, indicated that GMC levels are enhanced across years, but the level of competence and its development are strictly dependent on weight status during childhood. Our results provide strong evidence for objective gaps in GMC in overweight children with respect to normal-weight children, as well as in girl children compared to their boy peers, across school ages. The maintenance of normal weight status over late childhood and pre-adolescence allows good levels of GMC and a progressive increase in motor competence. Overweight status during childhood and pre-adolescence represents an adverse condition to the harmonious and normal development of gross motor coordination. Special attention is thus needed for pupils, especially those who do not practice sports.

We found that BMI value significantly affects GMC capacity within the majority of the classes monitored in this study and that overweight young people always show lower levels of GMC than normal-weight subjects, being unable to fill the gap in GMC competence with respect to their normal-weight counterparts throughout the years. Due to the high relevance of weight status on GMC, our results indicate the need not only to promote physical education to directly increase GMC levels but also to promote healthy habits in order to maintain adequate body weight and indirectly help GMC improvement in children. GMC assessment was suggested as an indicator of health status in children (32) and interventions based on GMC development are essential, in particular for overweight children, because of their high likelihood of also presenting lower GMC levels during early adolescence (58).

## Strengths and limitations of the study

Although longitudinal studies have many strengths, they are not free from inherent weaknesses. For example, it should be mentioned that the validity of longitudinal results is influenced by the regression to the mean, a phenomenon that affects research designs that use multiple measures to document changes in a variable over time. This could have influenced the results of the present study and could lead to interpreting a change over time as given by the factor investigated when it is just the regression to the mean. In particular, the trajectories of GMC for the OW and NW groups can be influenced in a different way due to this phenomenon. It should be noted, however, that measurement errors and the reliability of measures are key factors for regression to the mean, other than the degree to which a selected subgroup differs from the population mean (59). Given that the reliability of the KTK score used in our investigation has been reported to be very high, approximately 0.97 (16), the above-mentioned effect should be small. Although the KTK usually requires subjects to perform it barefoot, in this study, to ensure greater safety against the risk of slipping, participants were asked to wear shoes. This may have improved the scores of some tests but should not have affected the comparison between groups and the trend over time as the condition was maintained for all subjects and all years. A further limitation of this study is the small sample size for the first and last class investigated, as well as the absence of information about children’s sport participation and habits. The lack of BMI-GMC correlation significance in 3PS and 3SS could be due to a lower number of subjects evaluated in these classes.

## Data availability statement

The raw data supporting the conclusions of this article will be made available by the authors, without undue reservation.

## Ethics statement

The study involving human participants was approved by the Ethics Committee of the University of Verona (N. UNVRCLE-0298910). Written informed consent for participation in the study was provided by the participants' parents/legal guardians.

## Author contributions

VB, BP, CZ, FG, ML, MG, and FS: conceptualization. BP: formal analysis. VB, MG, and ML: methodology. VB: project administration. FS: supervision. VB, FG, and MG: writing-original draft. VB, FG, MG, CZ, FS, and BP: writing – review and editing. All authors contributed to the article and approved the submitted version.
